# Survival of patients with mucosal melanoma in Cali, Colombia: a retrospective cohort study

**DOI:** 10.1186/s12885-024-12371-x

**Published:** 2024-07-23

**Authors:** Ana M. García, Luis G. Parra-Lara, Diana M. Mendoza-Urbano, Juan C. Bravo, Ángela Zambrano Harvey

**Affiliations:** 1 Hematology and Clinical Oncology Service, Department of Internal Medicine, Fundación Valle del Lili, Cra. 98 Nro.18 -49, 760032 Cali, Colombia; 2 Centro de Investigaciones Clínicas (CIC), Fundación Valle del Lili, Cali, Colombia; 3 Facultad de Ciencias de La Salud, Universidad Icesi, Cali, Colombia; 4 Departament of Pathology and Clinical Laboratory, Fundación Valle del Lili, Cali, Colombia

**Keywords:** Mucosal melanoma, Neoplasm staging, Survival, Immunotherapy

## Abstract

**Background:**

Mucosa melanoma is a rare condition with aggressive behavior and a less favorable prognosis compared to cutaneous melanoma. The objective of this study was to estimate the overall survival and clinical outcomes of patients diagnosed with mucosal melanoma in a Colombian hospital.

**Methods:**

A retrospective cohort study was conducted at Fundación Valle del Lili, a single center located in Cali, Colombia. Patients aged ≥ 18 years, both sexes, diagnosed with mucosal melanoma by histopathology study were included between 2010–2019. Patients who received extra-institutional treatment or whose vital status was unknown during follow-up were excluded. Demographic, clinical and laboratory data were obtained from medical records and laboratory and pathology databases. A descriptive analysis was performed. Survival analysis was conducted using the Kaplan–Meier method.

**Results:**

A total of 23 patients were included. Median age was 63 years old (IQR: 57–68) and 52.2% were woman. Clinical stage was 34.8% early, 26.1% locally advanced and 39.1% metastatic. The main primary locations were nasopharynx (30.4%), genitals (26.1%), rectum (21.7%), oral cavity (13%) and paranasal sinuses (8.7%). The majority received surgery (30.4%) and immunotherapy (26.1%) as first line treatment.

Overall survival at one year was 80.8%, at three years 44.3%, and at five years 36.9%.

**Conclusion:**

Mucosal melanoma is a rare, aggressive disease with adverse oncological outcomes due to late diagnosis and limited treatment options. This study provides real-world data in a single-center of Colombia.

## Background

Melanomas are malignant tumors that arise from pigment cells and can arise from both skin and mucosal surfaces [1], being the second a rare condition. It is an aggressive cancer arising in melanocytes within ectodermal mucosa. However, the pathogenesis of mucosal melanoma is unknown and rarely carries the mutation in B-type Raf (BRAF), c-KIT (CD117), NRAS, GNAQ/11 and programmed death-ligand 1 (PD-L1) expression [1–3].

The National Cancer Database from the American College of Surgeons reported 1,074/84,836 (1.3%) cases as mucosal melanoma from cutaneous and noncutaneous melanoma database during period 1985–1993 in USA [4], and its most frequent locations correspond to the mucosal surfaces of the respiratory, gastrointestinal and genitourinary tracts, where melanocytes are present. Primary sites of origin include the head and neck (55%), anorectum (24%) and vulvovaginal region (18%) [5].

**Fig. 1:**
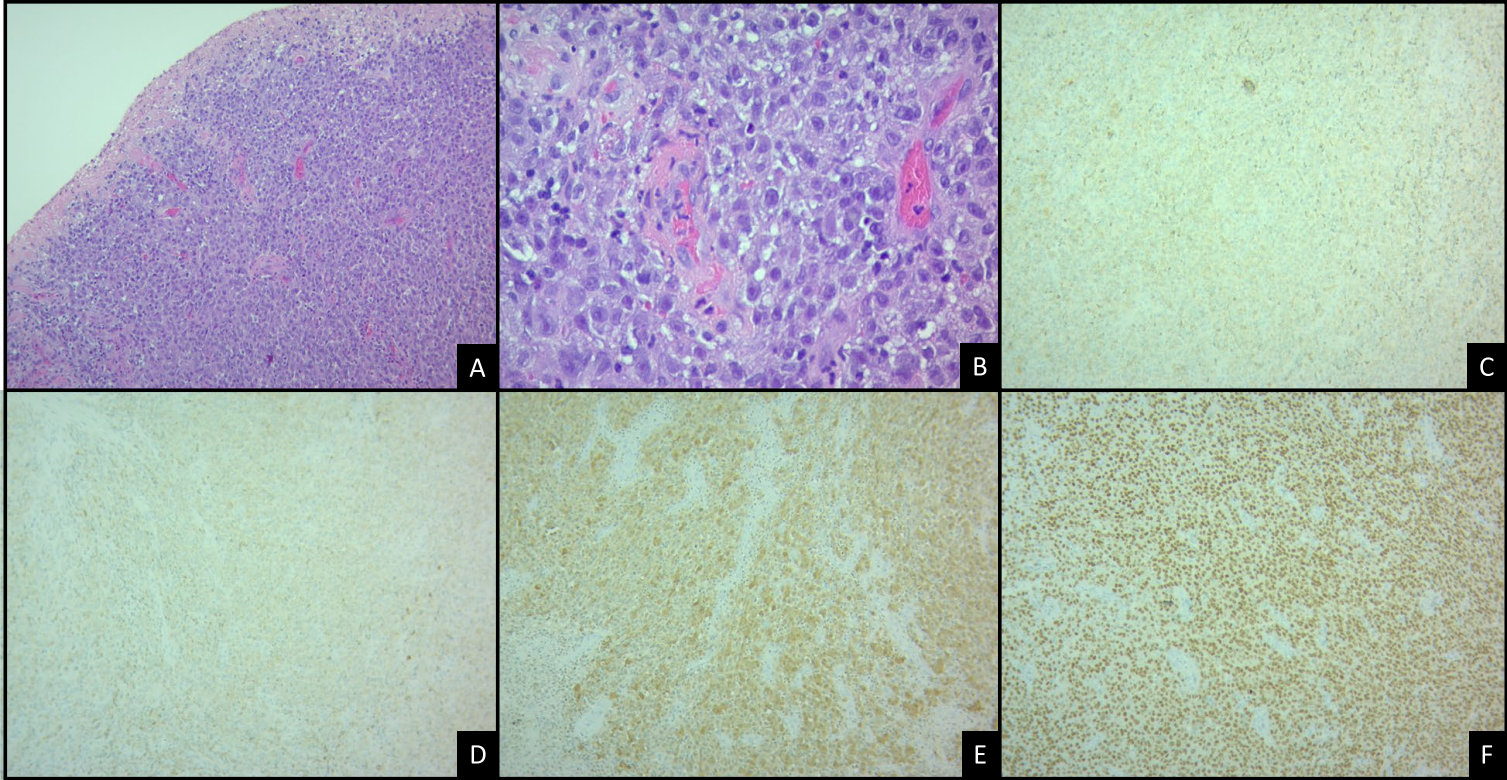
Invasive rectal melanoma arising in rectal mucosal of a 60 year-old woman. **A** Eroded-rectal mucosa occupied by sheets of epithelioid, pleomorphic malignant cells growing in a solid pattern (H&E 4X). **B** Lamina propia with mucosal melanoma cells showing high nucleus-to-cytoplasm ratio, hyperchromatic nuclei and prominent nucleoli (H&E 40X). Melanoma tumor cells immunohistochemical stains (10X) with MelanA (**C**) and HMB45 (**D**) cytoplasmic expression; S100 nuclear and cytoplasmic positivity (**E**); and SOX10 nuclear stain (**F**). *(Courtesy Juan Carlos Bravo*, *MD*, *Cali*, *Colombia)*

The tumour is usually composed of sheets or expansive nodules of large pleomorphic epithelioid or (less commonly) malignant melanocytic spindle cells [6, 7]. Pigmentation is variable and may be absent. Necrosis is rare. The nuclei often have vesicular chromatin and prominent nucleoli. Occasionally, small or naevoid cells may predominate. Less frequently, a lentiginous growth of individual atypical melanocytes in the basal layer may occur, sometimes with nests or confluent growth. A subepithelial lymphocytic infiltrate is common [8]. Figure 1 shows the histological examination of a clinical case of rectal melanoma.

In Colombia, the estimates on melanoma made by population-based registries exclusively include information on melanoma in general or cutaneous melanoma, which limits the epidemiological information on this neoplasm in the country [9–11]. However, it is known that this type of melanoma has an aggressive behavior and a less favorable prognosis compared to cutaneous melanomas [1]. The objective of this study was to estimate the overall survival and clinical outcomes of patients diagnosed with mucosal melanoma in a Colombian hospital.

## Materials and methods

### Study design and setting

A retrospective, hospital-based, observational cohort study was conducted in Cali, Colombia.

Cali, the capital of the Valle del Cauca Province, is the third city in the country, with around 2,250,000 inhabitants in 2019 [12]. During five-year period 2013–2017, 24,963 new cancer cases were diagnosed in permanent residents of Cali with an age-standardized incidence rate for all locations men were 191.2 and 175.4 in women. Age-standardized incidence rates of cutaneous melanoma per 100,000 person-year were 1.3 in men and 2.2 in women, and other skin neoplasms was 0.9 for both sexes [9].

Fundación Valle del Lili is a high-complexity university hospital that serves as a reference center in southwestern Colombia. It is one of the five hospitals that has integrated oncological services in the city, with a hospital-based cancer registry (HBCR) that includes data related to patient identification, cancer identification, the first course of treatment and outcomes. The case definition and registry methodology has been previously described [13]. Data extraction was done by active search and continuous.

The Registro Poblacional de Cáncer de Cali (RPCC) is a population-based cancer registry that has operated continuously since 1962. It includes the new cases of cancer throughout notification and active searching in primary data sources, including hospitals, clinics, pathology laboratories, and cancer centers in Cali. The RPCC has good information quality indicators [14, 15].

In the hospital, a total of 324 cases of melanoma have been identified in the period 2014–2018, based on the collaborative work between the HBCR and the RPCC, and these represent approximately 1.1% of all cancer cases treated during that period [13].

### Case definition and selection

Cases were obtained from the hospital database from 2010–2019. To identify the patients, an initial screening was performed using the International Classification of Diseases, 10th edition (ICD-10: C43) and subsequently the cases of mucosal melanoma were identified.

Patients aged ≥ 18 years old, both sexes, with a histopathological diagnosis of melanoma located in the mucosa were included.

### Matching

Mucosal melanoma cases were matched with cancer databases (HBCR and RPCC) to obtained clinical, pathology, follow-up and vital status data.

### Follow-up

Vital status and the date of death or the last follow-up day were determined using the cancer databases (RPCC or HBCR), general hospital mortality, hospital discharge, or the health system affiliation database (BDUA).

### Data

Retrospective data were obtained from hospital medical records and pathology reports. Demographic, tumor classification, staging, treatment, and follow-up variables were collected. The IARC/WHO International Classification for Diseases in Oncology, 3rd Edition (ICD-O-3) was used for topography and morphology. Clinical staging (cTNM) was done taking into account the AJCC Classification, 8th Edition [16].

Eastern Cooperative Oncology Group (ECOG) performance status was obtained to determine ability of patient to tolerate therapies (0: asymptomatic; 1: symptomatic but completely ambulatory; 2: symptomatic, < 50% in bed during the day; 3: symptomatic, > 50% in bed, but not bedbound; 4: bedbound; 5: death) [17, 18].

Lactic dehydrogenase (LDH) levels were obtained at diagnosis (U/L). Ki-67 was used as an indicator of cell proliferation. Mutational biomarkers BRAF and KIT were recorded from the histopathology and immunohistochemistry reports, if they had been performed. PDL-1 levels were also collected.

Initial treatment was defined as any therapeutic intervention against the neoplasm that was carried out immediately after diagnosis (surgery, radiotherapy, chemotherapy). Regarding chemotherapy, it was classified as conventional therapy (i.e., temozolamide, vinblastine, interferon, bevacizumab and/or paclitaxel) or immunotherapy (i.e., Pembrolizumab, ipilimumab y/o nivolumab).

Response to the treatment was assessed using response evaluation criteria in Solid Tumors 1.1 (RECIST) as complete response (CR), partial response (PR), progressive disease (PD) and stable disease (SD) [19].

Recurrence was defined as the appearance of any type of tumor lesion after the patient had presented a complete response.

Date of diagnosis was taken as the date of the pathology report.

The duplicate cases in every sources of registries were identified and removed. Some characteristics such as identification, date of birth, health insurance regime, residence, ICD-O-3 code related to their cancer type and vital status were used to identify the common cases among databases.

### Statistical analysis

A descriptive analysis was performed. Survival analysis was conducted using the Kaplan–Meier method. Survival was calculated using the date of diagnosis and the date of death or the last day of follow-up (the last day of hospital care and the date of last contact recorded; the most recent date was used). The reference date of last contact was taken as September 30, 2023. Overall survival for 12, 36 and 60 months of follow-up was calculated.

A value of *p* < 0.05 was considered statistically significant. All analyses were performed using STATA® (Version 14.0, StataCorp LP, College Station, TX).

## Results

A total of 23 patients were included according to the selection criteria. Table 1 shows the demographic and clinical characteristics of the included patients.

**Table 1:** Demographic and clinical characteristics of patients diagnosed with mucosal melanoma (*n* = 23)

Characteristic	n (%)
**Median age (IQR) – yr**	63 (57–68)
**Female sex**	12 (52.2)
**Clinical stage**	
Early	8 (34.8)
Locally advanced	6 (26.1)
Metastatic	9 (39.1)
**Topography of the primary lesion**	
Nasopharynx	7 (30.4)
Genitals	6 (26.1)
Rectum	5 (21.7)
Oral cavity	3 (13)
Paranasal sinuses	2 (8.7)
**ECOG**	
0	3 (13)
1	12 (52.2)
2	6 (26.1)
3	2 (8.7)
**Median LDH (IQR) – U/L** ^*^	176 (144–229)
**Median Ki-67 (IQR)—%** ^**†**^	40 (35–70)
**First line treatment**	
Surgery	7 (30.4)
Immunotherapy	6 (26.1)
Multimodal	5 (21.7)
Palliative care	5 (21.7)
**Response to first-line treatment**	
Complete response	9 (39.1)
Partial response	1 (4.3)
Stable disease	2 (8.7)
Progressive disease	9 (39.1)
Unknown	2 (8.7)
**Vital status**	
Death	17 (73.9)
Alive	6 (26.1)

*ECOG* Eastern Cooperative oncology group performance status scale, *IQR* Interquartile range, *LDH* Lactate dehydrogenase test

^*^ *n* = 15

^†^ *n* = 9

The age range was from 30 to 88 years, the majority were female (52.2%) and lived in an urban area (91.3%). The most frequent locations were nasopharynx (*n* = 7) and genital area (*n* = 6).

The clinical stage at diagnosis was: 13.1% stage I, 21.7% stage II, 26.1% stage III and 39.1% stage IV. Nine patients underwent testing for B-RAF, and all were unmutated. Four patients underwent testing for PDL-1, of which one was positive for 20% of tumor cells. There was one case in which KIT was performed and it was positive. A 56.5% of the patients debuted with an adequate functional status at the time of diagnosis (ECOG 0 and 1). Nine patients presented a CR to treatment, 66.6% (6/9) presented at least one relapse in clinical follow-up, and 86.9% (20/23) required management with a second treatment.

**Fig. 2:**
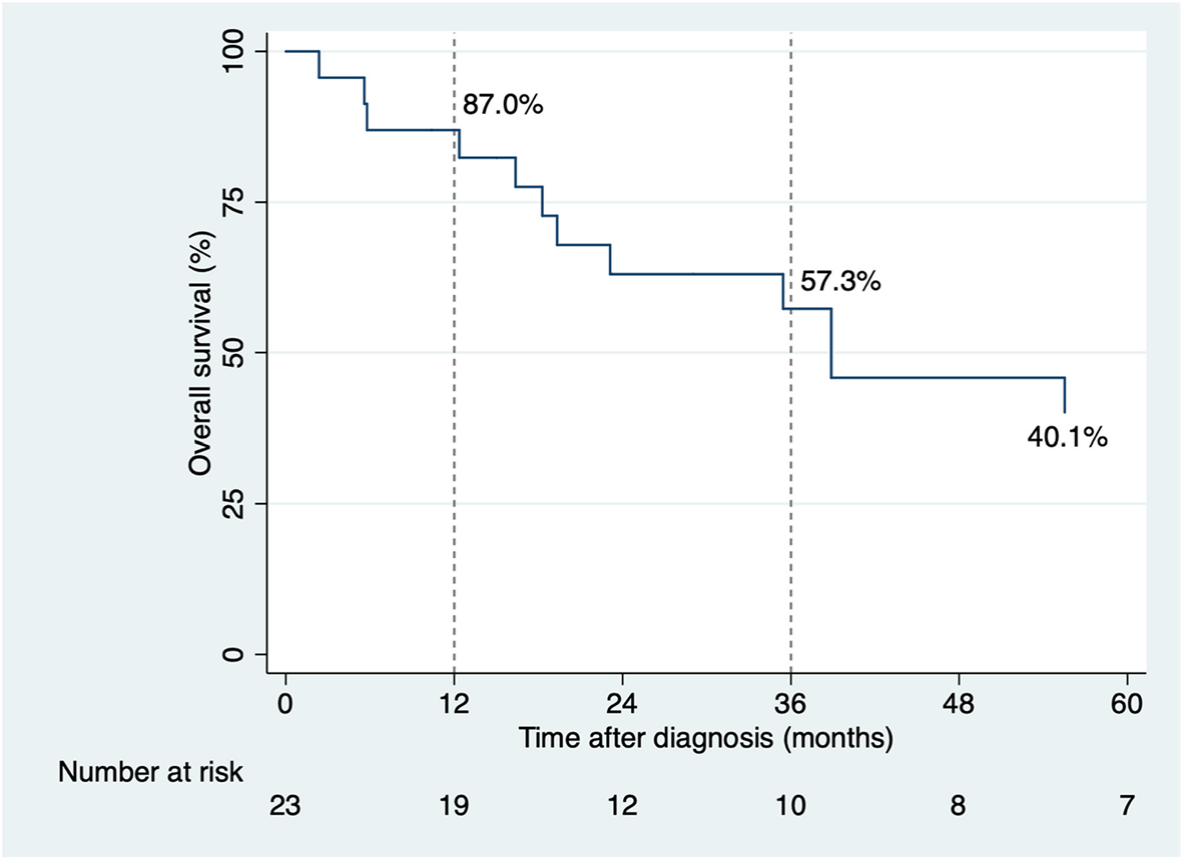
Overall survival of patients with mucosal melanoma (*n* = 23)

The median survival time was 38.9 months (95% CI, 18.3–80.5). Overall survival at one year was 87.0% (95% CI, 64.8–95.6), at three years 57.3% (95% CI, 33.5–75.3), and at five years 40.1% (95% CI 18.8–60.7). Figure 2 presents the survival curve.

## Discussion

Mucosal melanoma remains a rare disease and this study shows a panorama for the Latin American population. A 5-year overall survival was estimated at 40.1% in a hospital of the southwestern region on Colombia, being the first made in the country. However, despite advances in the treatment of cutaneous melanoma, patients with mucosal melanoma have limited benefit from currently available treatments because this disease has been little studied due to its rarity [20].

Mucosal melanoma has an epidemiological, clinical, and pathological behavior different from that of cutaneous melanoma [21]. Most mucosal melanomas occur in occult sites and do have not early and specific clinical signs, which contributes to a late diagnosis and poor prognosis [22]. This justifies clinical staging at the time of diagnosis, where we found that 65.2% were already in an advanced stage. Given the low frequency of this disease, the information is limited to retrospective studies carried out in countries such as China [23], Austria [24], Brazil [25], the United States [26, 27] and Iran [28].

The age of onset of mucosal melanoma is higher than cutaneous melanoma, with a mean age of 70 years old at diagnosis, while for cutaneous melanoma is approximately 55 years old [21]. In our study, the median age was 63 years old, showing that the onset presentation in our population is earlier than the literature shows, which is why it should be alert in this age group at the time of diagnostic suspicion.

Regarding sex, mucosal melanoma occurs more frequently in women [21, 29]. In them, the most frequent commitment is vulvovaginal, while in men, the most affected area is the head and neck [22, 29]. In our study, the main locations of mucosal melanoma were the vulva, rectum and maxillary sinuses, while in men it was in the nasal cavity, genitourinary tract and nasopharynx, which is related to the descriptions made in other studies.

Treatment of mucosal melanoma continues to be a challenge, due to the anatomical location of the primary lesions, lentiginous and multifocal growth, which makes a complete resection with negative margins difficult, generating a high recurrence rate after surgical resection or performing surgeries with high morbidity [20]. The role of adjuvant treatment is also not entirely clear; on the one hand, radiotherapy has shown limited benefit in local control, without achieving an impact on overall survival [30, 31]; on the other hand, chemotherapy has effects similar to those known in cutaneous melanoma, without being able to demonstrate a significant improvement in outcomes [20]. In our study, the main therapeutic strategy was surgical resection, but since most of the cases showed an advanced stage and a significant tumor size, the management options also included a multimodal strategy or palliative care.

Immunotherapy data is limited due to the rarity of this disease and the exclusion of this melanoma subtype from clinical studies. Consequently, the available data has been obtained from small subgroup analyzes or retrospective studies [31]. Results showed that mucosal melanoma has a lower response to immune checkpoint inhibitors compared to cutaneous melanoma, which could be explained because mucosal melanoma is less immunogenic than cutaneous melanoma [20].

The implementation of immunotherapy in our country for mucosal melanoma began in 2013 when these therapies were approved by regulatory entities for the treatment of cutaneous melanoma. That is why 26.1% of cases received this treatment option. Immunotherapy offers clinical benefit in patients with limited treatment options and within this option, combined immunotherapy has shown better response rates [31]. In a retrospective pooled analysis of patients with unresectable or metastatic melanoma who received nivolumab or ipilimumab in monotherapy or in combination, it was found that in patients with mucosal melanoma the combination of nivolumab and ipilimuab had better progression-free survival compared to immunotherapy in monotherapy [32].

Our 5-year overall survival was 40.1%, considerably higher than the 25% described in the United States for the period 1985–1989 [4]. This can be explained by several points: [1] the observation period, which was different in both studies (our study had cases diagnosed between 2008–2018), [2] the sample size, it was smaller in our study and could overestimate survival and [3] the availability of systemic treatment strategies, these include immunotherapies and targeted treatments. Also, they stratified by location, 5-year survival was 31.7% for head and neck, 11.4% for the female genital tract, and 19.8% for anorectal, with a better prognosis for head and neck locations (*p* < 0.05), but a worse outcome in those with the presence of lymphatic involvement (16.4% vs. 38.7%) [4].

### Limitations

Due to the study design, selection and information bias could be introduced into the results. First, our university hospital is a referral center for the treatment of cancer patients with advanced stages, which could explain the proportion of patients with this clinical stage at diagnosis, although it is known that due to the characteristics of this neoplasm, most cases have a late diagnosis. Second, the sources of information were based on the retrospective review of medical records and institutional databases (laboratory and pathology), which could affect the data collection such as information related to immunohistochemistry and biomarkers because not all histopathology studies were performed at the hospital (fragmentation of Colombian health system).

However, to improve the data quality, our database was cross-linked with the population-based cancer registry (RPCC, Universidad del Valle) database, a high-quality cancer registry. This, in order to improve data related to the vital status and follow-up. Finally, because it is a rare disease, the sample size was not sufficient to perform regression models that would allow exploring factors associated with survival in this population.

## Conclusions

Mucosal melanoma is a rare, aggressive disease with adverse oncological outcomes due to late diagnosis and limited treatment options. This study provides real-world data in a single-center of Colombia.

## Data Availability

The datasets used and/or analysed during the current study are available from the corresponding author on reasonable request.

## Abbreviations

AJCC: American joint committee on cancer
CR: Complete response
ECOG: Eastern cooperative oncology group
IARC: International agency for research on cancer
ICD: International classification of diseases
IRB: Institutional review board
LDH: Lactic dehydrogenase
PD: Progressive disease
PR: Partial response
RECIST: Response evaluation criteria in solid tumours
RIC: Institutional cancer registry
RPCC: Cali cancer registry
SD: Stable disease
WHO: World health organization
